# Selenium requirements based on muscle and kidney selenoprotein enzyme activity and transcript expression in the turkey poult (*Meleagris gallopavo*)

**DOI:** 10.1371/journal.pone.0189001

**Published:** 2017-11-30

**Authors:** Rachel M. Taylor, Roger A. Sunde

**Affiliations:** Department of Nutritional Sciences, University of Wisconsin, Madison, Wisconsin, United States of America; Leibniz-Institut fur Pflanzengenetik und Kulturpflanzenforschung Gatersleben, GERMANY

## Abstract

The current NRC selenium (Se) requirement for turkeys is 0.2 μg Se/g diet. We previously fed turkey poults a Se-deficient diet (0.005 μg Se/g) supplemented with 10 graded levels of Se (0, 0.025, 0.05, 0.1, 0.2, 0.3, 0.4, 0.5, 0.75, 1.0 μg Se/g as Na_2_SeO_3_, 5/treatment) for 4 wk, and found that the minimum dietary Se requirement was 0.3 μg Se/g based on selenoprotein enzyme activity in blood, liver, gizzard and pancreas. Because the turkey is primarily a production animal, we expanded this analysis to kidney, heart, breast and thigh. Se concentrations in Se-deficient poults were 5.0, 9.8, 33, and 15% of levels in poults fed 0.4 μg Se/g in liver, kidney, thigh and breast, respectively. Increasing Se supplementation resulted in hyperbolic response curves for all tissues; breakpoint analysis indicated minimum Se requirements of 0.34–0.36 μg Se/g based on tissue Se levels in liver, kidney and thigh. Similarly, GPX1 activity in muscle tissues and kidney responded hyperbolically to increasing dietary Se, reaching well-defined plateaus with breakpoints at 0.30–0.36 μg Se/g. Minimum Se requirements based on GPX4 activity were 0.30–0.32 μg Se/g for breast and thigh. Selenoprotein transcript expression decreased significantly in Se deficiency for only 2, 3, 5, and 6 mRNA in breast, thigh, heart, and kidney, respectively, out of 24 known avian selenoproteins. Se response curves for regulated selenoprotein transcripts were hyperbolic, and reached well-defined plateaus with breakpoints in a narrow range of 0.08–0.19 μg Se/g. No selenoprotein transcript was altered by supernutritional Se. In summary, these results clearly indicate that the NRC dietary Se requirement should be raised to 0.4 μg Se/g, at least for poults, to meet the nutritional needs of the young turkey. The Se response curve plateaus further show that limits for turkey supplementation with selenite could safely be raised to 0.5 μg Se/g diet.

## Introduction

The current NRC Se requirement for turkeys at all stages of growth and production is 0.2 μg Se/g diet [1], based on studies conducted 40–50 years ago on growth and protection against gizzard myopathy [1,2]. More recent studies indicate that the Se requirement of modern, rapidly-growing commercial turkeys is 0.3 μg Se/g diet, based on glutathione peroxidase (GPX) activities in plasma, liver and gizzard as biomarkers [3,4]. Recently, we fed day-old poults 10 graded levels of Se as selenite, from Se-deficient (0.005 μg Se/g) to 1.0 μg Se/g for four weeks, and determined a minimum dietary Se requirement of 0.3 μg Se/g diet, based on blood, liver and gizzard GPX activities [5]. These recent studies, along with changes in modern strains of commercial turkeys [6], suggest that there is a need to better characterize the Se requirements of the turkey.

Turkeys are the 4th most economically-significant food animal species (after swine, bovine, and chicken); turkey production in the United States totaled $5.7 billion in 2016 [7]. Strains used today have been heavily selected for growth and muscle development [8]. Meat can be a major source of Se in human diets, but the FDA currently limits Se supplementation in livestock feeds to a maximum of 0.3 μg Se/g diet out of concern for adverse effects of excess Se intake [9]. The European limit is 0.5 μg Se/g diet for inorganic selenium [10]. Because of these concerns and the commercial emphasis on meat production, muscle Se biomarkers should also be considered when setting dietary Se requirements for the turkey.

Turkey Se requirements based on selenoenzyme activities are at least three times higher than Se requirements in mammals [11]. Studies in rats have shown that selenoprotein transcripts can be used as biomarkers for Se status and requirements [12,13]. In our recent study, we also studied Se regulation of selenoprotein transcript levels in turkey liver, gizzard and pancreas, and found that minimum Se requirements were lower than requirements based on selenoenzyme activity [5], just as in rodents. Turkeys do not have genes for three Se-containing proteins found in higher animals (glutathione peroxidase 6, selenoprotein V, and selenophosphate synthase 2, but express selenoprotein U (SELENOU) and a second plasma selenoprotein P2 (SELENOP2); turkeys also express the non-Se-containing selenophosphate synthase 1 (SEPHS1) [14]. Analysis of selenoprotein transcripts in the turkey may help to explain differences between avian and mammalian Se requirements, and may provide additional biomarkers for adequate vs. excess Se intake, useful in a reconsideration of the FDA limit for Se supplementation.

We have now expanded analysis for GPX1 and GPX4 activities and selenoprotein transcript expression to breast, thigh, heart, and kidney from our previous study, and for Se concentrations in breast, thigh, kidney, and liver. Our objectives were: 1) to determine minimum Se requirements based on tissue Se concentration, selenoenzyme activity, and transcript expression in these tissues; 2) to characterize the impact of additional dietary Se above the Se requirement on these biomarkers; 3) to identify potential biomarkers of high Se status.

## Methods

This manuscript adopts the new systematic nomenclature of selenoprotein names [15].

### Reagents

Molecular biology reagents were purchased from Promega (Madison, WI, USA), Invitrogen (Carlsbad, CA, USA), or Sigma (St. Louis, MO, USA). All other chemicals were of molecular biology or reagent grade.

### Animals and diets

Day-old male Nicholas white-derived turkey poults (kindly donated by Jennie-O Turkey Store, Barron, WI) were allocated randomly to treatment and housed in battery cages (5–6 per cage) with raised wire floors and 24-hr lighting, as described previously [5]. The basal Se-deficient torula yeast-based diet contained 0.005 μg Se/g as described previously [5], with all-rac-α-tocopherol acetate at 150 mg/kg (12.5X NRC requirement [1]) to prevent vitamin E-deficiency related disease. The basal diet was supplemented with ten graded levels of Se (0, 0.025, 0.05, 0.1, 0.2, 0.3, 0.4, 0.5, 0.75, 1.0 μg Se/g diet) as Na_2_SeO_3_. Body weight was measured twice weekly and feed intake per cage was measured weekly. The animal protocol was approved by the Research Animal Resources Committee at the University of Wisconsin-Madison (protocols no. A01146 and A005368).

### Tissue collection

The poults (5 per treatment) were killed at 28 days by terminal CO_2_ overexposure followed by exsanguination. Liver, breast muscle (pectoralis major), thigh muscle (iliotibialis), heart and kidney were collected and immediately frozen at -80°C until analysis. On tissue collection, one bird from the 0 μg Se/g diet group was found to be female and was omitted from statistical analysis.

### Enzyme activity analysis

Kidney, heart, breast and thigh were analyzed for GPX4 activity using the coupled assay procedure [5,16] with 78 μmol/L phosphatidylcholine hydroperoxide (PCOOH). GPX1 was assayed as described previously [5,17] with 120 μmol/L H_2_O_2_, and GPX1 specific activity was calculated by subtracting the activity due to GPX4, as described previously [13]. In both assays, 1 enzyme unit is defined as the amount of enzyme that will oxidize 1 μmol glutathione per min under the specified conditions. The protein concentration was determined by the method of Lowry et al. [18].

### Tissue Se analysis

Neutron activation analysis was conducted on liver, kidney, thigh, and breast (n = 3/treatment) at the University of Missouri Research Reactor Center to determine Se concentrations [19], and expressed as nmol Se/g wet weight. For 22 of 30 thigh samples, a fatty fraction separated upon freeze drying. Tissue Se was determined separately for the fat and lean fractions, and total Se concentration calculated based on fractional weights.

### RNA isolation and analysis

Total RNA from kidney, heart, breast and thigh (n = 5/group) was isolated using TRIzol Reagent (Invitrogen, catalog no. 15596–026) as described previously [5]. Relative mRNA abundance was determined by quantitative real-time PCR (qPCR). Turkey gene-specific primer sets were based on the recently sequenced turkey selenoproteome [14] and designed to span an intron-exon splice junction and amplify a 120-150-base segment. The final 12 μl reactions contained 2 μl reverse transcribed cDNA working stock, 0.2 mmol/L turkey gene-specific forward and reverse primers, and 1X KAPA SYBR FAST qPCR Kit (KAPA Biosystems no. KK4611). Reactions were followed in a LightCycler 480 (Roche Life Science). Melting curves were generated to confirm the presence of one specific product and standard curves were run for each primer set/tissue combination, and the second derivative max program was used to determine amplification efficiency following the manufacturer’s protocol. mRNA relative abundance was calculated according to Pfaffl [20], accounting for gene-specific efficiencies, normalized to the mean of β-actin (ACTB) and glyceraldehyde-3-phosphate dehydrogenase (GAPDH) expression, and expressed as a percentage of the plateau of Se-adequate levels. To compare transcript expression of different selenoproteins, relative abundance was normalized for basepair length of the amplified fragment.

### Statistical analysis

Data are presented as mean ± SEM. For growth, enzyme analysis and mRNA expression, n = 5, except in the 0 μg Se/g diet group (n = 4); for tissue Se concentration, n = 3. Data were analyzed by one-way ANOVA and variance equality was tested using Levene’s test for homogeneity of variances [21]. When the main effect of diet was significant, differences between means were assessed by Duncan’s multiple range test (P<0.05) with Kramer’s modification for unequal class sizes when necessary [22]. A “Se-response curve” was constructed using sigmoidal or hyperbolic regression analysis (Sigma Plot, Jandel Scientific) using all individual values at each dietary Se treatment, as described previously [5,12]. The “plateau breakpoint” for each Se response curve, defined as the intersection of the line tangent to the point of steepest slope and the plateau, was calculated as described previously [5,12] to estimate the minimum dietary Se requirement necessary to obtain the plateau response. For biomarkers that continued to increase with increasing dietary Se after a breakpoint, the plateau linear regression line was calculated using individual values for treatments on the plateau that were not significantly different by the ANOVA.

## Results

We report here kidney, heart, breast, and thigh selenoprotein enzyme activities and transcript expression, and Se concentrations in kidney, breast, and thigh as well as liver in poults supplemented with 0–1.0 μg Se/g diet for 4 weeks. For this study, we previously reported selenoprotein enzyme activity and transcript expression in plasma, RBC, liver, gizzard and pancreas of the poults; that analysis clearly showed that poults fed the basal diet were Se deficient, and that 0.3 μg Se/g diet was the minimum dietary Se requirement poults for turkey poults [5]; the summary data from that report has been included in **Table 1** for comparison. Because biomarker levels for poults fed 0.4 μg Se/g reside on the plateaus of the Se response curves, we have designated 0.4 μg Se/g as the Se-adequate treatment for comparison purposes.

**Table 1:** Se requirement hierarchy in growing poults.

**Traditional Biomarker**	**Requirement**^**a**^	**Regulation**^**b**^	***P-value***
	**(μg Se/g diet)**	**(%)**	
Final body weight	<0.005	n/a	0.6100
Growth rate	0.05	63.2	2.29E-05
Liver TXNRD activity*	0.07	18.0	1.37E-06
Gizzard GPX1 activity*	0.18	18.0	0.0190
Heart GPX4 activity	0.18	29.7	4.80E-10
Liver GPX4 activity*	0.23	6.0	1.46E-17
Gizzard GPX4 activity*	0.24	10.4	2.63E-05
Pancreas GPX4 activity*	0.25	34.3	5.84E-05
Kidney GPX4 activity	0.25	10.0	3.00E-12
Breast Se	0.27	16.3	1.90E-08
Plasma GPX3 activity*	0.29	1.1	9.30E-12
RBC GPX1 activity*	0.29	45.1	2.44E-10
Pancreas GPX1 activity*	0.30	0.1	0.0054
Kidney GPX1 activity	0.30	1.5	2.61E-15
Thigh GPX4 activity	0.30	32.9	5.14E-07
Breast GPX4 activity	0.32	7.9	5.49E-09
Breast GPX1 activity	0.32	9.4	3.55E-05
Liver GPX1 activity*	0.33	4.1	5.09E-16
Heart GPX1 activity	0.34	20.5	3.06E-09
Thigh Se	0.34	32.8	1.60E-06
Thigh GPX1 activity	0.35	0.4	3.10E-08
Kidney Se	0.35	9.7	1.40E-14
Liver Se	0.36	6.3	2.12E-12
**Transcript Biomarker**	**Requirement**	**Regulation**	***P-value***
Liver DIO1 mRNA*	0.05	32.1	0.1500
Liver SELENOP1 mRNA*	0.05	36.0	0.1800
Gizzard SELENOH mRNA*	0.06	33.6	0.0024
Liver GPX1 mRNA*	0.07	34.8	0.0388
Liver SELENOH mRNA*	0.07	19.5	0.0419
Gizzard GPX3 mRNA*	0.07	40.9	0.0020
Heart SELENOH mRNA	0.08	31.5	0.0001
Liver GPX3 mRNA*	0.08	35.3	0.0245
Liver GPX4 mRNA*	0.08	29.4	0.1323
Liver SELENOU mRNA*	0.09	29.3	0.0396
Thigh GPX4 mRNA	0.10	73.4	0.0211
Breast GPX1 mRNA	0.11	46.9	0.0132
Breast SELENOH mRNA	0.11	47.7	0.0020
Kidney DIO1 mRNA	0.13	39.0	0.0003
Kidney SELENOH mRNA	0.13	44.2	0.0040
Kidney SELENOP1 mRNA	0.13	37.3	0.0024
Thigh SELENOH mRNA	0.13	48.6	0.0005
Heart GPX1 mRNA	0.13	31.4	3.52E-06
Pancreas SELENOP1 mRNA*	0.13	57.0	0.0289
Kidney GPX3 mRNA	0.14	48.4	0.0016
Thigh GPX1 mRNA	0.14	58.5	0.0078
Heart SELENOP1 mRNA	0.14	62.7	0.0050
Heart GPX3 mRNA	0.14	50.0	0.0179
Gizzard GPX1 mRNA*	0.14	46.0	3.82E-05
Gizzard GPX4 mRNA*	0.15	46.2	0.0111
Pancreas SELENOH mRNA*	0.15	34.8	0.0191
Kidney GPX4 mRNA	0.16	51.1	0.0048
Heart GPX4 mRNA	0.18	54.1	0.0345
Pancreas GPX3 mRNA*	0.18	29.3	0.0001
Kidney GPX1 mRNA	0.19	38.5	0.0258

^a^Minimum dietary Se requirement as determined by breakpoint analysis

^b^Extent of regulation: Percentage of Se-deficient as compared to 0.4 μg Se/g diet group

*Se requirements reported previously for liver, gizzard, and pancreas [5]

### Growth and feed efficiency

As previously reported for these poults [5], there was no significant effect of dietary Se supplementation on final body weight (P = 0.61, **S1 Table**), but poults fed 0 and 0.025 μg Se/g diet had average weights that were 23% and 17% lower, respectively, than the weight average of the other groups. The 0 and 0.025 μg Se/g diet groups did have significantly lower rates of growth from day 7 to day 28 that were 36% and 25% less, respectively, than the average growth of the other Se supplementation groups [5]. The minimum Se requirement based on growth rate was 0.05 μg Se/g diet (**Table 1**).

Reported here, weekly treatment-group feed conversion ratios (FCR) were calculated by dividing the weekly feed consumption by the weekly total bodyweight gained per treatment. The average FCR for weeks 1, 2, and 3 were 1.2±0.02, 1.8±0.04, and 1.8±0.09, respectively. For week 4, the average FCR was 2.2±0.08 for poults supplemented with Se, whereas poults fed 0 μg Se/g diet had an FCR of 3.31; the lowest week 4 FCR of 1.7 was for poults fed 0.4 μg Se/g diet. Upon dissection, all tissues appeared grossly normal in these poults supplemented with 150 mg/kg vitamin E (12.5X NRC requirement[1]). Other details of the study have been previously reported [5].

### Tissue Se concentration

**Fig 1:**
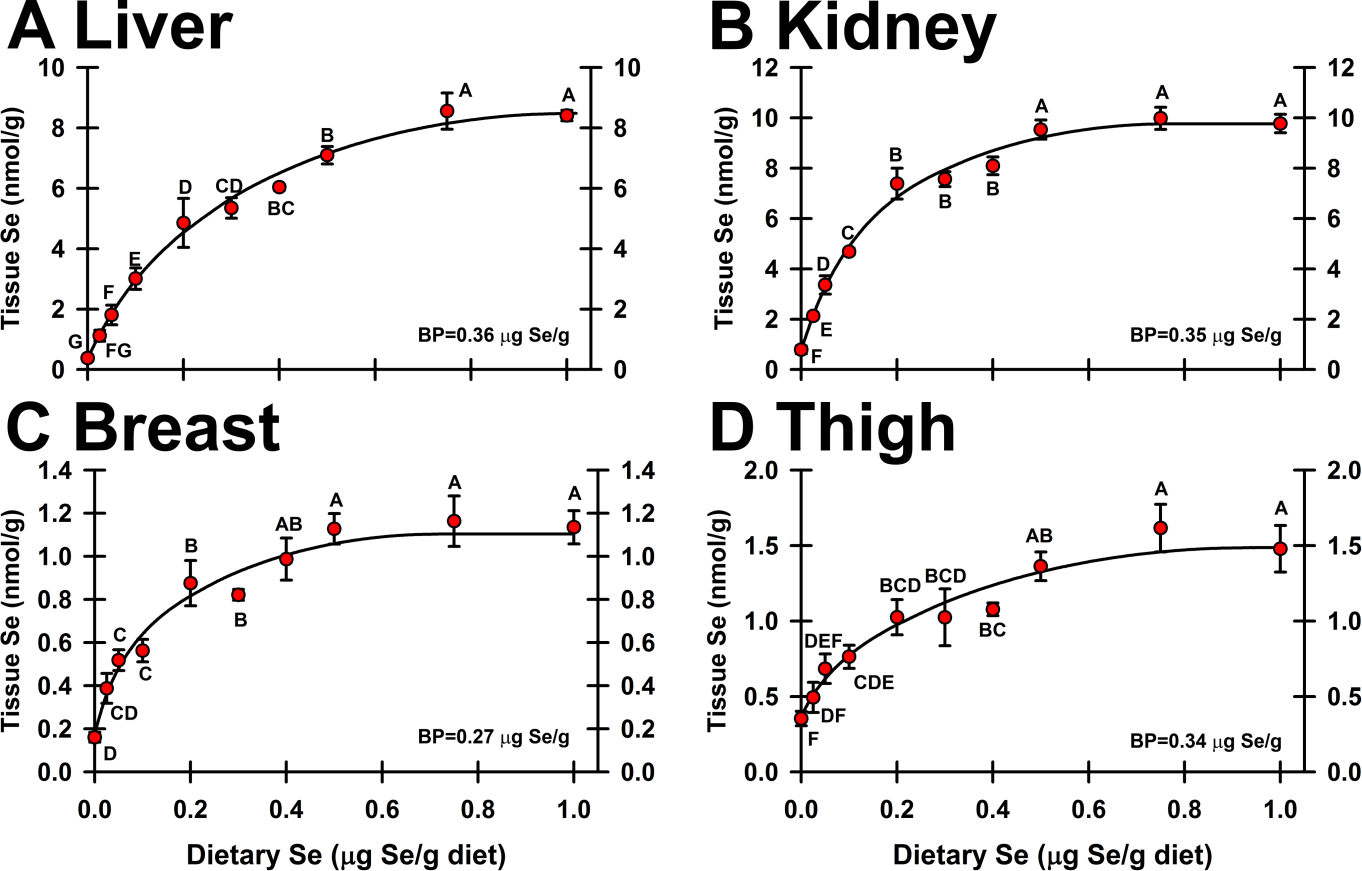
Effect of dietary Se on tissue Se concentration. Se concentration in liver (A), kidney (B), breast (C), and thigh (D), expressed as μg Se/g wet tissue. Values are means ± SEM (n = 3/treatment). Means without a common letter are significantly different (p<0.05). Overall levels of significance, as determined by ANOVA, are given in **Table 1**. Se response curve breakpoints (BP) are calculated as described in text.

In poults fed the Se-adequate level of 0.4 μg Se/g diet, Se concentrations were 6.80 ± 0.41 nmol Se/g in liver, 8.10 ± 0.35 nmol Se/g in kidney, 1.10 ± 0.04 nmol Se/g in thigh, and 1.10 ± 0.04 nmol Se/g in breast. Feeding the Se-deficient diet for 4 wk resulted in tissue Se concentrations that were 5.0%, 9.8%, 33% and 15%, respectively, of Se-adequate levels (**Fig 1**). Supplementation with 1.0 μg Se/g diet only increased tissue Se concentrations 39%, 21%, 40%, and 15% above levels in poults fed 0.4 μg Se/g diet, indicating a plateauing of tissue Se level in response to increasing levels of dietary Se above 0.4 μg Se/g diet.

The Se response curves for Se concentrations (**Fig 1**) were hyperbolic, with the steepest increase in tissue Se between 0 and 0.2 μg Se/g diet, and with clear plateauing at ≥ 0.5 μg Se/g diet. For liver (**Fig 1A**), plateau breakpoint of the Se response curve was 0.36 μg Se/g diet (**Table 1**), and the Se response curve breakpoint in kidney was 0.35 μg Se/g diet (**Fig 1B**). For thigh, the Se response curve breakpoint was 0.34 μg Se/g diet (**Fig 1C**). These minimum Se requirements are similar to those we reported in these poults based on GPX1 activities in liver and pancreas. For breast, the Se response curve breakpoint (**Fig 1D**) was lower, at 0.27 μg Se/g diet, and higher than minimum Se requirements based on GPX4 activities in liver, gizzard and pancreas, and on GPX1 activities in gizzard in these poults (**Table 1**).

### Enzyme activity

**Fig 2:**
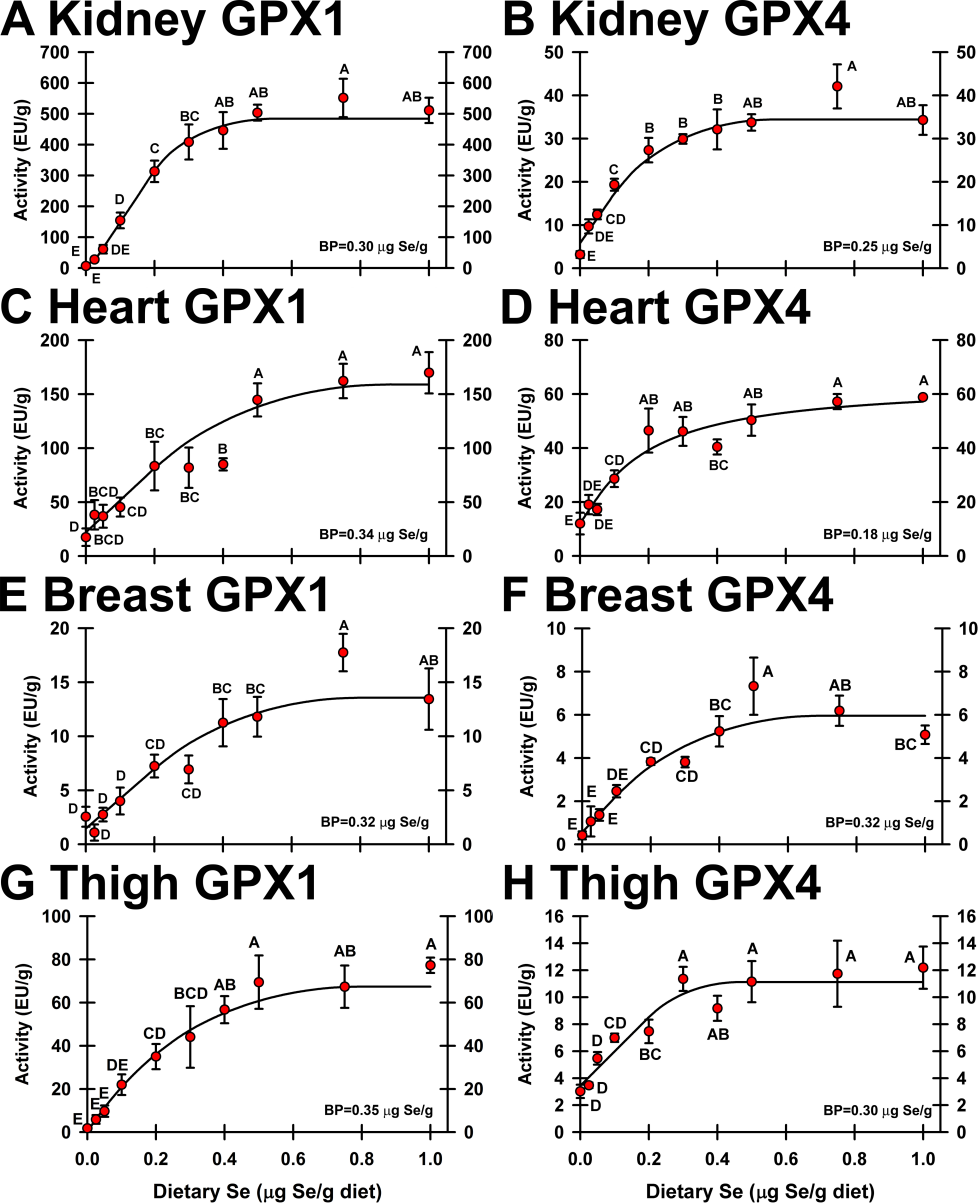
Effect of dietary Se on GPX activity. GPX1 and GPX4 activity in kidney (A, B), heart (C, D), breast (E, F) and thigh (G, H). Values are the means ± SEM (n = 5/treatment). Means without a common letter are significantly different (p<0.05). Overall levels of significance, as determined by ANOVA, are given in **Table 1**. Se response curve breakpoints (BP) are calculated as described in text.

The relative rank of GPX1 activity in poults fed 0.4 μg Se/g diet was kidney >> heart > liver, RBC >thigh > gizzard, pancreas, breast (**Fig 2**). GPX1 activities in poults fed the Se-deficient diet were 1.5%, 21%, 0.4% and 9.4% of Se-adequate levels in kidney, heart, thigh and breast, respectively, further demonstrating that these poults were Se-deficient. Increasing dietary Se resulted in a sigmoidal Se-response curve for kidney GPX1 activity (**Fig 2A**), with a plateau breakpoint at 0.30 μg Se/g diet. The Se-response curves for GPX1 activity in heart, thigh and breast (**Fig 2B–2D**), were hyperbolic, with plateau breakpoints of 0.30, 0.33, and 0.32 μg Se/g diet, respectively. Se supplementation with 1.0 μg Se/g diet only significantly increased GPX1 activity above levels in 0.4 μg Se/g diet poults in heart, further showing that supernutritional Se does not significantly increase tissue GPX1 activity.

GPX4 activity was measured using PCOOH as a substrate. The relative rank of GPX4 activity in poults fed 0.4 μg Se/g diet was liver > heart > kidney > thigh, gizzard > breast, pancreas (**Fig 2**). GPX4 activities in poults fed the Se-deficient diet were 10%, 30%, 33% and 7.9% of Se-adequate levels in kidney, heart, thigh and breast, respectively, showing that GPX4 activity was less impacted by Se deficiency than was GPX1 activity. Hyperbolic Se-response curves were observed for GPX4 activity in all tissues reported here (**Fig 2**) as well as for liver, gizzard and pancreas reported previously [5], with a plateau breakpoints at 0.25, 0.18, 0.30 and 0.32 μg Se/g diet for kidney, heart, thigh and breast, respectively. Se supplementation with 1.0 μg Se/g diet did not significantly increase Se concentration in these four tissues above levels in poults fed 0.3–0.4 μg Se/g diet, showing that supernutritional Se above the minimum Se requirement does not significantly increase tissue GPX4 activity, just as for GPX1 activity.

### Transcript expression

**Fig 3:**
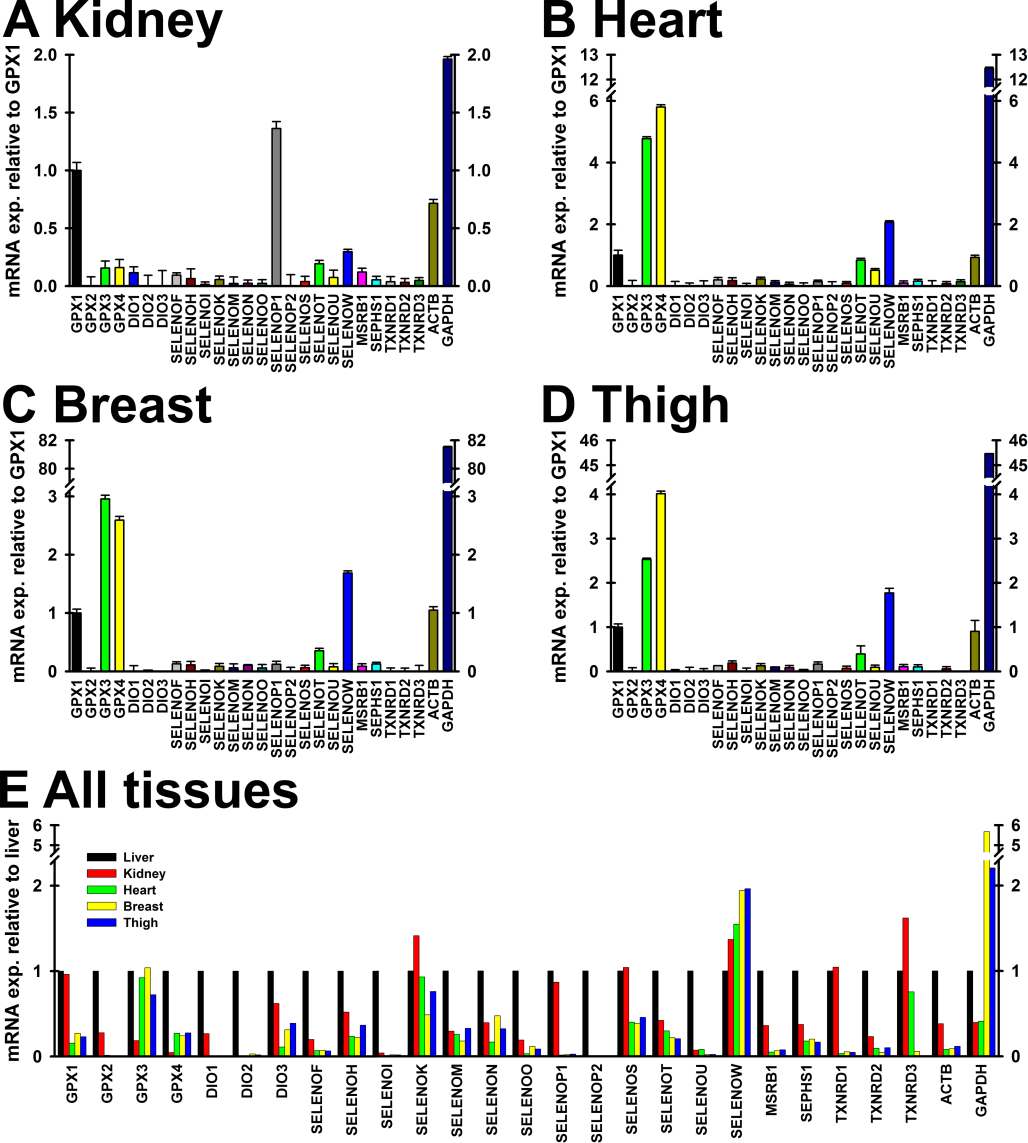
Transcript expression in Se-adequate poults. Transcript expression in poults fed 0.4 μg Se/g diet (n = 5) is plotted relative to GPX1 transcript expression in each tissue (A-D), and plotted relative to expression of each transcript in Se-adequate liver (E). Bars show means ± SEM (A-D).

To begin to evaluate the abundance of selenoprotein transcripts in kidney, heart, breast and thigh, an initial qPCR analysis was conducted for poults fed 0, 0.4 and 1 μg Se/g diet (n = 4, 5 and 5 per treatment, respectively). To compare the relative expression of the 24 selenoprotein transcripts, plus selenophosphate synthetase 1 (SEPHS1) and the housekeeping genes ACTB and GAPDH, the transcript levels were compared in poults fed 0.4 μg Se/g diet, and plotted relative to GPX1 expression (**Fig 3A–3D**). In kidney, GPX1 was the second-most abundant transcript, and selenoprotein P1 (SELENOP1) was expressed at 1.5X GPX1 transcript expression level. The 22 other selenoprotein genes and SEPHS1 were expressed at <0.5X the level of GPX1 expression (**Fig 3A**).

Muscle tissues all showed similar selenoprotein transcript expression patterns (**Fig 3B–3D**). GPX3 and GPX4 were expressed at 5-6X the level of GPX1 transcript expression in heart, 2-3X the level of GPX1 transcript expression in breast, and 2-4X the level of GPX1 transcript expression in thigh. In these muscle tissues, selenoprotein W (SELENOW1) was expressed at 1.5-2X the level of GPX1 expression, and Selenoprotein T (SELENOT) expressed at ~0.5X the level of GPX1 transcript expression. All other transcripts were expressed at <0.5X the level of GPX1 transcript expression. DIO1, DIO3, GPX2 and SELENOP2 transcripts were not expressed at detectable levels in heart, DIO1, GPX2, SELENOP2 and TXNRD3 transcripts were not expressed at detectable levels in breast, and GPX2 and SELENOP2 transcripts were not expressed at detectable levels in thigh.

**Fig 3E** shows the same data for each selenoprotein transcript expressed relative to the level in liver. Liver had the highest expression of any tissue for 18 of the 24 selenoprotein transcripts. GPX1 mRNA was highly expressed in liver and kidney. GPX3 mRNA was approximately equally expressed in all tissues, excluding kidney where it was expressed at <0.5X the level of liver expression. SELENOW1 mRNA was expressed at 1.5-2X the level of liver expression in kidney, heart, breast and thigh. SELENOK mRNA in kidney was expressed at 1.5X the level of liver expression. SELENOP1 was expressed at approximately the same level in liver and kidney but expressed at low levels in the other tissues; SELENOP2 was expressed at detectable levels in liver only.

### Transcript regulation in Se-deficient and high-Se poults

**Fig 4:**
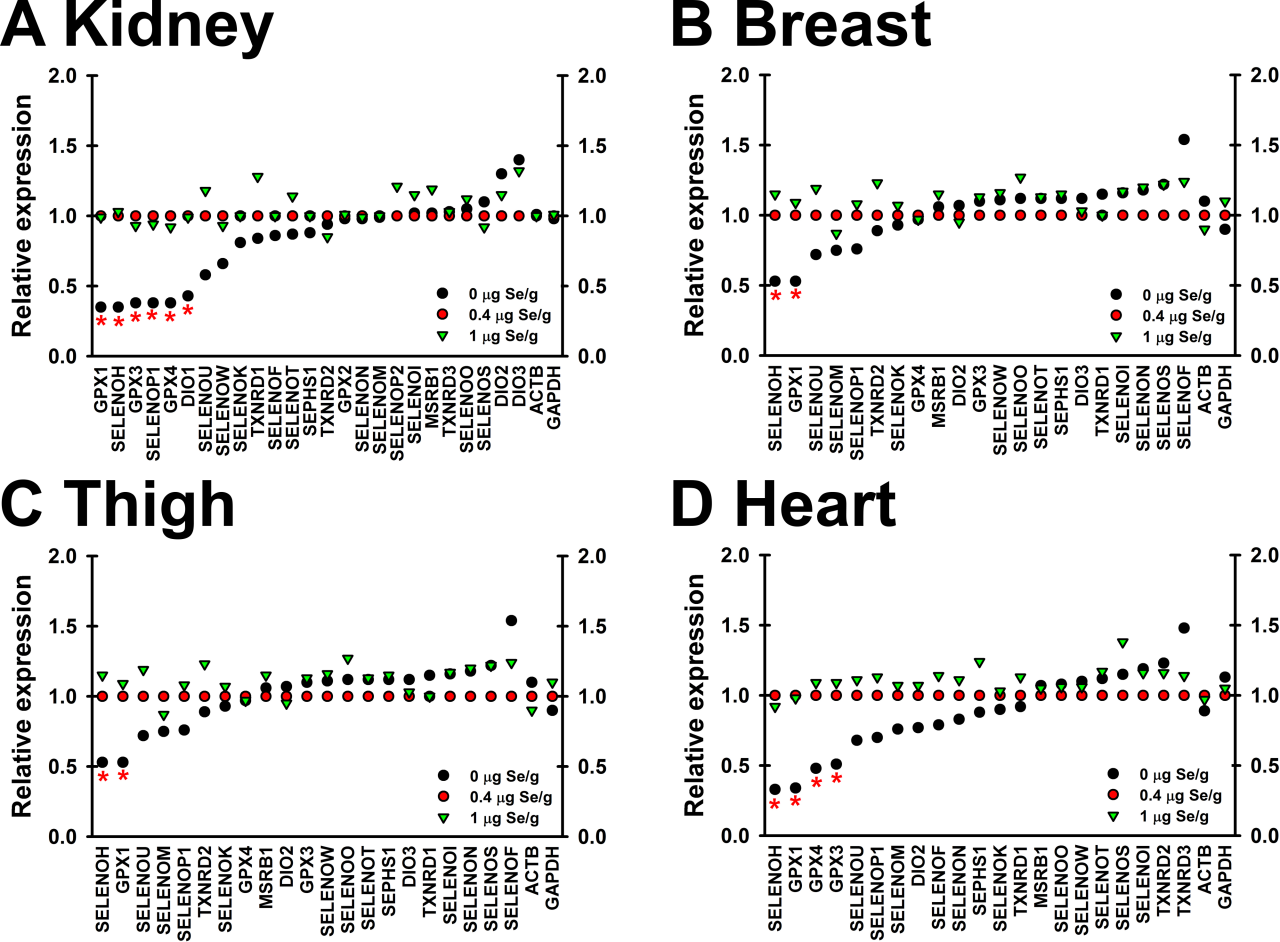
Effect of dietary Se on differential expression of selenoprotein transcripts. Transcript expression in kidney (A), breast (B), thigh (C), and heart (D) in poults supplemented with 0, 0.4 or 1 μg Se/g diet (n = 5/treatment), expressed relative to its level in Se-adequate (0.4 μg Se/g) poults. Asterisks indicate transcripts for which the effect of dietary Se was significant (p<0.05), as described in text.

To screen for selenoprotein transcripts potentially up- or downregulated by dietary Se status, transcripts from Se-deficient, Se-adequate and supernutritional Se treatments (0, 0.4 and 1 μg Se/g diet, respectively) were plotted relative to their expression in Se-adequate poults (**Fig 4**). Per manufacturer’s recommendations, transcripts that returned a raw Cp value within 2 cycles of the no-template control Cp values were considered not expressed at detectable levels, and were excluded from analysis. In this initial screen, 11 of the 24 selenoprotein transcripts in kidney were up- or downregulated ≥20% as compared to expression in Se-adequate poults. In heart, breast and thigh, 10 of 21, 7 of 21 and 10 of 22 expressed transcripts, respectively, were up- or downregulated ≥20% by dietary Se status (**Fig 4**).

**Fig 5:**
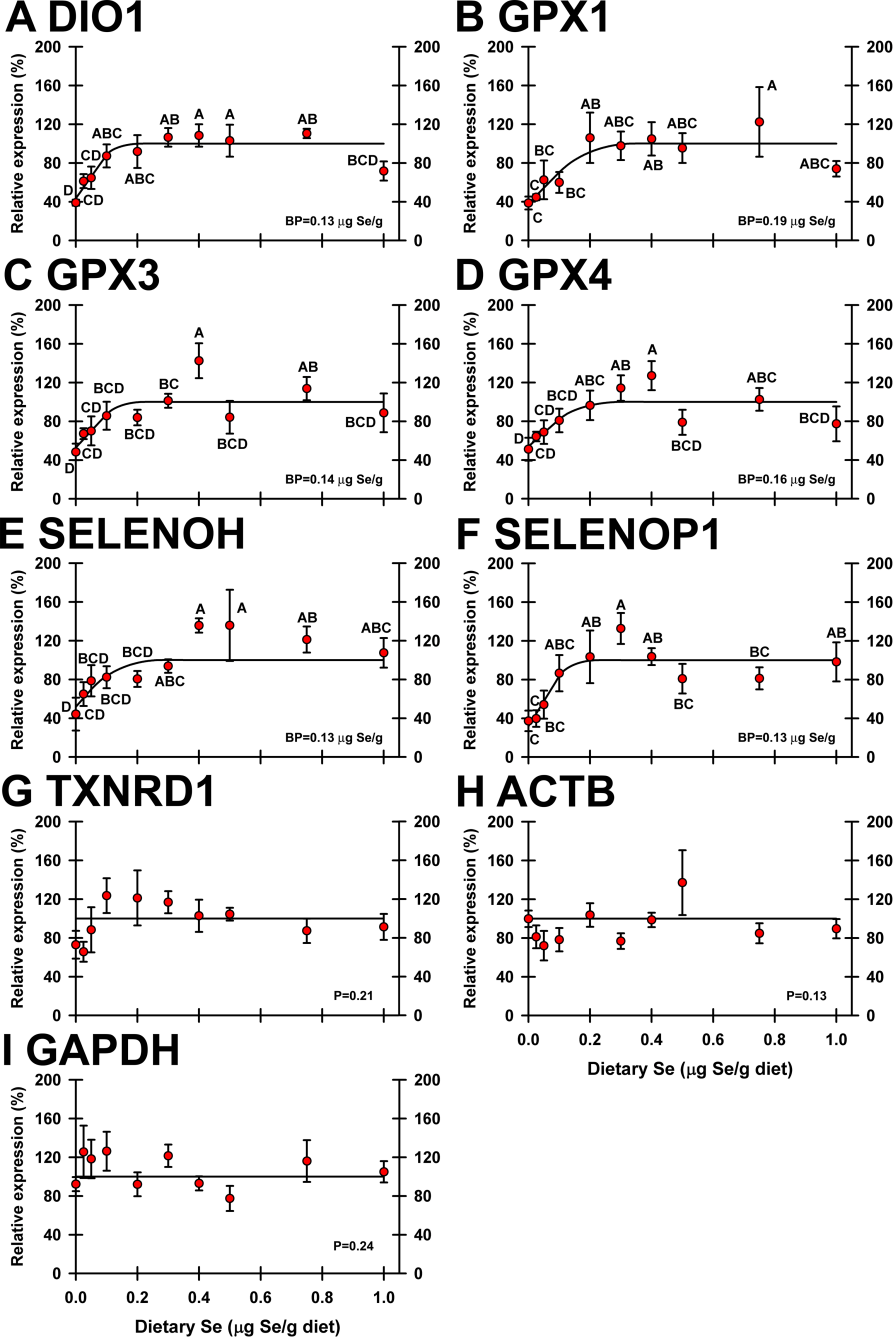
Effect of dietary Se on significantly-regulated selenoprotein transcripts in turkey kidney. Plotted are relative transcript levels for DIO1 (A), GPX1 (B), GPX3 (C), GPX4 (D), SELENOH (E), and SELENOP1 (F) in poults supplemented with graded levels of dietary Se for 28 d. Values shown are means ± SEM (n = 5/treatment). Means without a common letter are significantly different (p<0.05). Overall levels of significance, as determined by ANOVA, are given in **Table 1**. Se response curve breakpoints (BP) are calculated as described in text. Panels G and I show examples of transcripts (ACTB, GAPDH) not significantly regulated by dietary Se.

For transcripts identified as altered ≥20% by dietary Se status in the initial screen, qPCR was performed on all 10 Se treatments. In kidney, 6 of 24 selenoprotein transcripts (DIO1, GPX1, GPX3, GPX4, SELENOH and SELENOP1, and identified with asterisks in **Fig 4**) were found to be significantly downregulated by dietary Se deficiency (**Fig 5).** Se-response curves for all regulated kidney mRNA were hyperbolic and reached well-defined plateaus. In Se-deficient poults, selenoprotein transcript expression was 38–51% of plateau expression levels, with plateau breakpoints at 0.13–0.19 μg Se/g diet. Important as well, no selenoprotein mRNA in kidney was significantly regulated by supernutritional Se status. Also shown in **Fig 5**is the Se-response curve for TXNRD1 as well as ACTB and GAPDH as examples of transcripts not significantly regulated by dietary Se (**Fig 5G–5I**).

**Fig 6:**
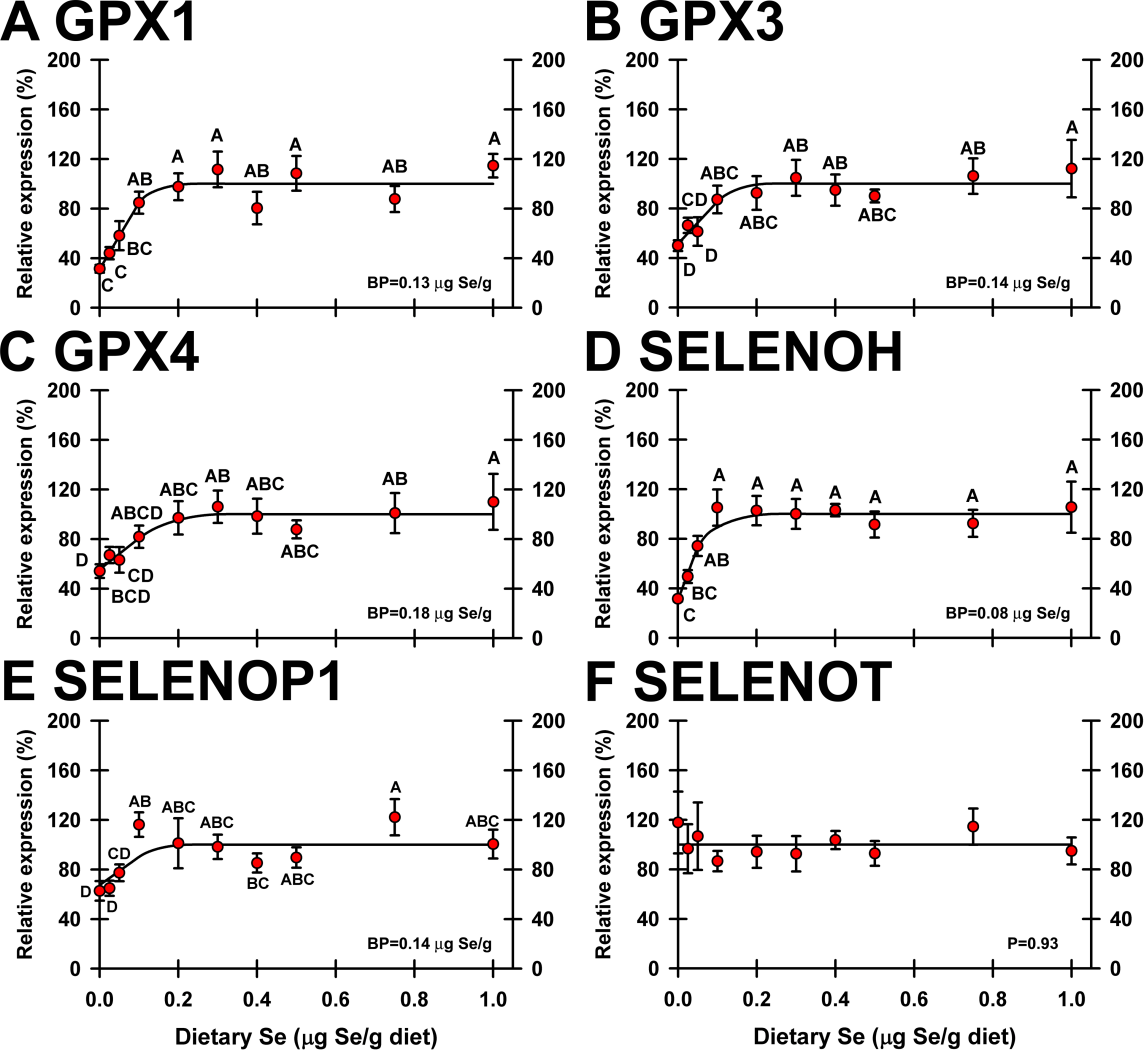
Effect of dietary Se on significantly-regulated selenoprotein transcripts in turkey heart. Plotted are relative transcript levels for GPX1 (A), GPX3 (B), GPX4 (C), SELENOH (D), SELENOP1 (E), and SELENOT (F) in poults supplemented with graded levels of dietary Se for 28 d. Values shown are means ± SEM (n = 5/treatment). Means without a common letter are significantly different (p<0.05). Overall levels of significance, as determined by ANOVA, are given in Table 1. Se response curve breakpoints (BP) are calculated as described in text. SELENOT is an example of transcript expression not significantly regulated by dietary Se.

In heart, 5 selenoprotein transcripts (GPX1, GPX3, GPX4, SELENOH and SELENOP1) were significantly downregulated by Se deficiency (**Fig 6**). Se response curves for these 5 selenoprotein mRNA were hyperbolic and reached well-defined plateaus. In Se-deficient poults, expression of the 5 regulated transcripts decreased to 31–63% of plateau expression levels, and plateau breakpoints were at 0.08–0.18 μg Se/g diet. No selenoprotein transcripts in heart were significantly regulated by supernutritional Se status.

**Fig 7:**
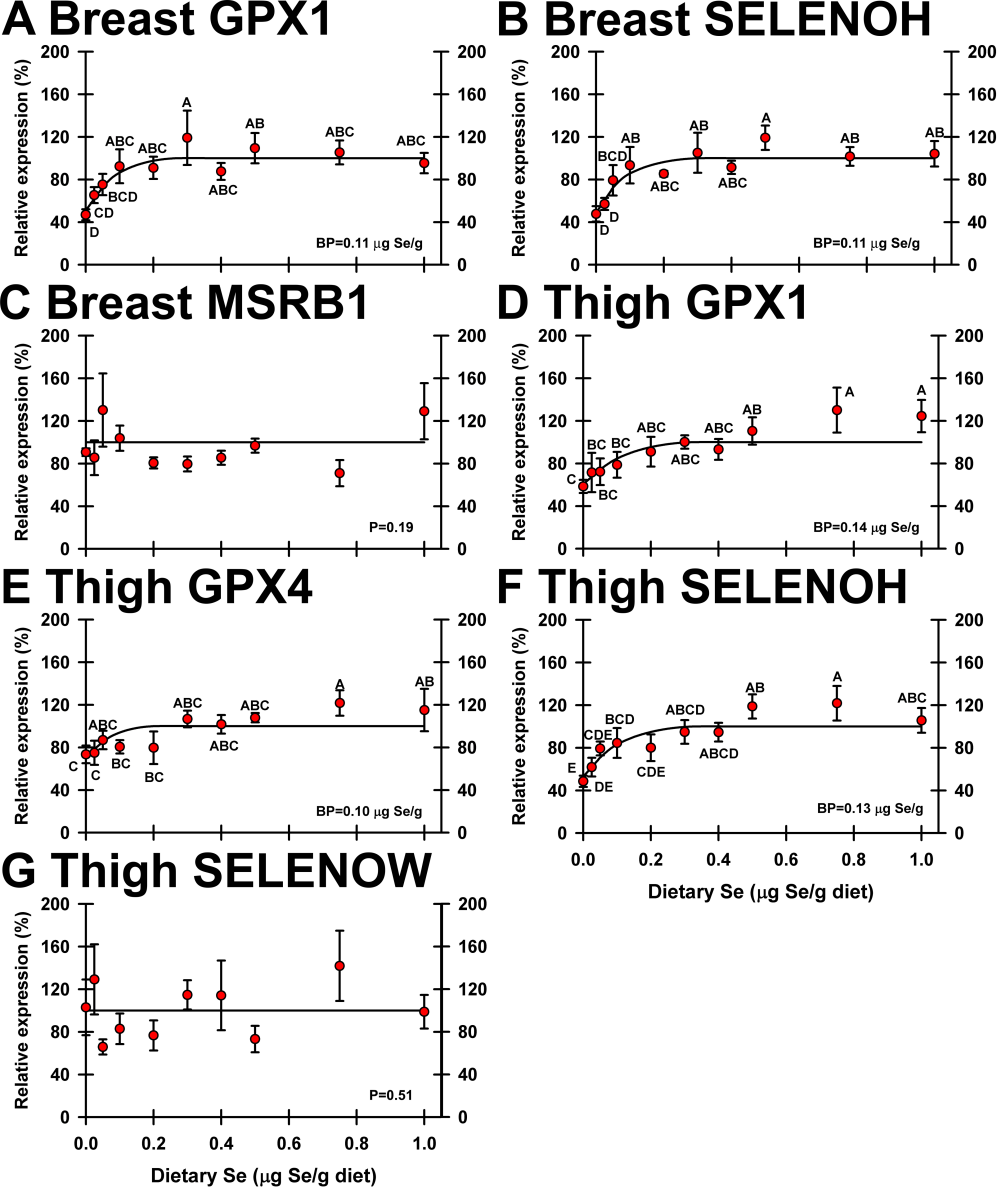
Effect of dietary Se on significantly-regulated selenoprotein transcripts in turkey breast and thigh muscle. Plotted are relative transcript levels for breast GPX1 (A), breast SELENOH (B), breast MSRB1 (C), thigh GPX1 (D), thigh GPX4 (E), thigh SELENOH (F), and thigh SELENOW1 (G) in poults supplemented with graded levels of dietary Se for 28 d. Values shown are means ± SEM (n = 5/treatment). Means without a common letter are significantly different (p<0.05). Overall levels of significance, as determined by ANOVA, are given in Table 1. Se response curve breakpoints (BP) are calculated as described in text. Breast MSRB1 and thigh SELENOW1 are examples of transcript expression not significantly regulated by dietary Se.

In breast and thigh, 2 and 3 selenoprotein transcripts were significantly downregulated by Se deficiency, respectively (**Fig 7A–7E**). As in the other tissues, Se response curves for these regulated selenoprotein transcripts were hyperbolic with well-defined plateaus. GPX1 and SELENOH mRNA decreased to 47 and 48% of plateau expression, respectively, in breast, with plateau breakpoints both at 0.11 μg Se/g diet. In thigh, GPX1, GPX4 and SELENOH mRNA expression was significantly downregulated by dietary Se deficiency to 49–73% of plateau expression levels (**Fig 7C–7E**), with plateau breakpoints at 0.10–0.14 μg Se/g diet. Just as in other tissues, no selenoprotein transcripts in breast or thigh muscle were significantly up- or down-regulated by supernutritional Se status.

## Discussion

The effects of dietary Se concentration on the Se response curves for 2 growth biomarkers, 4 tissue Se biomarkers, 17 selenoenzyme biomarkers, and 30 selenoprotein transcript biomarkers are summarized in **Table 1**. New analysis presented here reports that the minimum Se requirements were 0.27–0.36 μg Se/g, with requirements based on thigh, liver and kidney Se concentration greater than 0.3 μg Se/g. Minimum Se requirements based on muscle and kidney GPX1 activity were 0.30–0.34 μg Se/g whereas requirements based on GPX4 activities in these tissues were slightly lower, at 0.18–0.32 μg Se/g. Based on the analysis of muscle and kidney tissue Se and selenoenzyme activity, these results show that 0.3 μg Se/g is not sufficient to raise these biomarkers to plateau levels, strongly indicating that the Se requirement for the growing turkey poult should be raised to 0.4 μg Se/g, at least when inorganic Se is used.

The current NRC requirement for turkeys is 0.20 μg Se/g [1], in poults supplemented with vitamin E [2]. The recent reports [3–5] have shown that the Se requirement of turkey poults is 0.3 μg Se/g. Using Se response curves and graphical analysis, Fisher reported that 0.3 μg Se/g in male poults fed corn-soy diets was required to reach maximum levels of tissue Se in liver, plasma, gizzard and breast, and of plasma GPX3 activity [4]. In our earlier study, conducted with male poults using 30% torula yeast supplemented with just 6% additional amino acids, we found minimum Se requirements in gizzard and liver of 0.28–0.30 μg Se/g based on GPX1 and GPX4 activities; requirements based on heart, kidney and plasma enzyme activities ranged from 0.19–0.25 μg Se/g [3]. In these studies and the present study [5], poults were supplemented with adequate vitamin E. Clearly, the minimum requirement in young poults is greater than the current NRC requirement of 0.20 μg Se/g.

A second important feature of these recent studies [3–5] is the use of multiple graded levels of dietary Se. The study of Fisher et al. used 8 graded Se supplementation levels ranging from 0 to 0.4 μg Se/g with a basal diet containing <0.010 μg Se/g [4]. The study of Sunde and Hadley provided 7 supplementation levels ranging from 0 to 0.5 μg Se/g in a basal diet containing 0.007 μg Se/g [3], and the present study provided 10 grade levels from 0 to 1.0 μg Se/g [5]. The resulting Se response curves show that the highest rate of increase occurred between 0 and 0.2 μg Se/g for tissue Se and for selenoenzyme activity, and for the Se-regulated selenoprotein transcripts. For virtually all biomarkers, these curves further show that these biomarkers reach clear plateau levels over these dietary Se ranges, indicating that there are not substantial further increases in the tissue Se burden above the breakpoint. Clearly, homeostatic mechanisms are in place that limit tissue accumulation of Se when diets contain ≤ 1.0 μg Se/g, at least when inorganic Se supplements are used.

This study is one of the first to evaluate the effect of dietary Se on the expression of the selenoprotein transcripts in the turkey. Previous studies from our group and others have shown that there is a hierarchy of Se regulation of selenoprotein mRNA levels in rodents, chicks, and other species [11,12,16,23–25]. The mechanisms underlying this regulation, however, are not well understood and seem to be affected by a number of factors [13,26–28]. Some transcripts, such as Gpx1, fall dramatically in Se deficiency in rodents whereas other transcripts, such as Gpx4 mRNA, are minimally downregulated by Se deficiency [16]. Levels of the regulated selenoprotein transcripts thus can be used to identify Se deficiency, and we have shown that transcript expression can also be used as a biomarker for Se status and requirements [29]. In rodents, the minimum Se requirement based on the transcript expression was uniformly less than the requirement based on selenoprotein activity [12,13]. In the present study with turkey poults, the minimum Se requirements based on transcript expression were also lower than those based on selenoenzyme activity, ranging from 0.10–0.14 μg Se/g for breast and thigh, 0.08–0.18 μg Se/g for heart, and 0.13–0.19 μg Se/g for kidney enzyme activity. Generally, transcript-based requirements were highest for GPX1 or GPX4 transcripts, which in turn were associated with relatively high requirements for GPX1 and GPX4 enzyme activity.

In this study, only 5, 5, 3, and 2 transcripts, out of 24 turkey selenoproteins, were significantly regulated by Se status in heart, kidney, thigh and breast, respectively. We previously reported that only 4, 4, and 3 transcripts were significantly down regulated in liver, gizzard, and pancreas, respectively [5]. In a study with chicks conducted in parallel with the present study, we also found that only 8, 6, and 12 were significantly regulated in liver, gizzard, and pancreas, respectively [23]. In contrast, a number of recent studies in chicks reported that almost all selenoprotein transcripts were significantly regulated by Se deficiency [30–35]. These studies, however, typically only used a basal diet and one additional level of Se supplementation, easing the conditions for significance as compared to studies with multiple levels of supplementation. Furthermore, these studies often did not provide supplemental vitamin E, thus combining the effects of Se deficiency and vitamin E deficiency. In studies reporting that the levels of most selenoprotein transcript are significantly affected by Se status, the lack of a second level of protection against reactive oxygen species may have potentiated additional disease that contributed to the observed decreases for most of the selenoprotein transcripts.

The recent studies with turkey poults indicated that the Se requirement should be 0.3 μg Se/g [3–5], and the analysis presented here indicates that 0.4 μg Se/g is needed to support maximal levels of muscle Se and GPX activity. We conducted a parallel study in chicks, with virtually the same diets, and found that the minimum Se requirement for the growing chick was 0.15 μg Se/g, but that pancreas GPX activities suggested that the chick Se requirement should be increase to 0.2 μg Se/g [23]. Using the same experimental conditions, including use of Se-deficient (<0.01 μg Se/g) torula-yeast diets and selenite supplementation at multiple graded levels of Se, we found rats, mice and lambs have minimum Se requirements of just 0.1 μg Se/g. Use of this common experimental design shows that turkeys have dietary Se requirements 3-4X higher than for mammals, and that broiler chick Se requirements are 1.5-2X higher than for mammals [11].

The biology underlying the Se requirement differences between avians and mammals is not known, but may reside in differences in selenoprotein expression. Se absorption is high in avians as well as mammals [36], so this is not likely to be the major cause. Plateau levels of liver GPX1 activity in turkeys are 1/10^th^ the level in rodent liver, whereas plateau levels of liver GPX4 activity in turkeys are ~6 times the levels in rodent liver [3]. In Se deficiency, liver GPX1 activity falls to <10% of plateau levels in avians and rodents, but liver GPX4 activity falls to 10% of plateau levels in the turkey whereas it falls only to ~50% in rodents, showing clear differences in selenoprotein expression and regulation between avians and rodents [11]. In rats, liver Gpx1 mRNA levels fall dramatically to 10% of Se-adequate levels but Gpx4 mRNA levels are little affected [12] whereas in turkeys GPX1, GPX4 and Se-regulated transcripts decrease somewhat uniformly to 30–40% of Se-adequate levels [3,5]. In mammals, plasma SELENOP1 is the major Se transport protein, synthesized primarily in the liver, and targeted to testes and brain, mediated by SELENOP1-specific receptors [37]. In turkeys, SELENOP1 mRNA is highly expressed in kidney as well as liver [5], consistent with the kidney being a major source of circulating SELENOP1. SELENOP1 expression is low in avian muscle tissues. Avians have a second selenoprotein P, SELENOP2, not found in mammals, and SELENOP2 is highly expressed in turkey liver at levels similar to ACTB [5]. The present study shows that GPX3 transcripts are highly expressed in turkey muscle and heart but not kidney, and our previous analysis showed high GPX3 expression in gizzard comparable to GAPDH expression [5]. In mammals, plasma GPX3 is expressed and secreted primarily from the kidney [38]. These differences likely underlie some of the differences between avians and mammals in Se requirements and Se metabolism, and differences in development of Se deficiency diseases.

One of the objectives of this study was to examine the effect of supernutritional dietary Se supplementation and to identify potential biomarkers for excess Se. When rats were supplemented with up to 0.8 μg Se/g, we did not identify any biomarkers for high Se status [12]. In the present study as well, the Se response curves (**Fig 2**) clearly show that once sufficient Se is provided, there is little further increase in selenoenzyme concentrations; only heart GPX1 and GPX4 activities were significantly lower in poults fed 0.4 μg Se/g vs. at least one of 0.5, 0.75 or 1.0 μg Se/g treatments. Furthermore, there were no transcripts that were altered significantly up or down by 0.5, 0.75 or 1.0 μg Se/g as compared to 0.4 μg Se/g, including expression of the transport selenoproteins SELENOP1 and SELENOP2. This suggests that avians do not adjust to higher intakes of Se by altering expression of selenoprotein expression. Just as we have done in rodents [12,13,39] and in C. elegans [40,41], we are exploring expression of selenoproteins and non-selenoproteins at higher levels of Se supplementation to identify potential biomarkers for high and toxic Se status in turkeys.

Currently, FDA Title 21 U.S. Code, Sec. 573.920 Selenium, states that Se can be added as sodium selenite, sodium selenate, or selenium yeast “In complete feed for chickens, swine, turkeys, sheep, cattle, and ducks at a level not to exceed 0.3 part per million” [9]. This regulation was enacted so that animals were not consuming unsafe levels of the nutrient, so that unsafe levels of the nutrient were not consumed by individuals from edible portions of animals that received Se, and because Se above such levels “does not achieve its intended effect of promoting normal growth and reproduction of livestock and poultry.” Previous reports show that, at least in the male turkey poult, the current NRC Se requirement of 0.2 μg Se/g should be increased [3–5]. The data presented here indicates that the NRC Se requirement should be increased to 0.4 μg Se/g so that plateau levels of Se and Se-dependent enzymes are maintained. In addition, the data clearly demonstrate that tissue Se concentrations are maintained at plateau levels in poults fed 0.4 to 1.0 μg Se/g in breast, thigh and kidney. Relative to the FDA limit, the increase in dietary Se from 0.3 to 1.0 μg Se/g (a 3.33-fold increase) in this study was only accompanied by 33%, 44%, 29%, and 57% increases in Se concentration in turkey breast, thigh, kidney and liver, respectively; for all these tissues, the mean Se concentration in turkeys fed 1.0 μg Se/g was nominally less than that in turkeys fed 0.75 μg Se/g. These results thus demonstrate that exceeding the FDA limit of 0.3 μg Se/g as inorganic selenium has but modest impact on tissue Se concentrations, indicating that the homeostatic mechanisms in the turkey minimize storage of additional tissue Se over the range of 0.3 μg Se/g to 1.0 μg Se/g when Se is supplied as inorganic Se.

Early studies in turkeys found that selenite and SeMet were equivalent in preventing gizzard myopathy and raising GPX activity; poults fed SeMet had higher Se concentrations in gizzard, breast, and pancreas but not liver and heart [42]. There are no recent studies in turkeys that carefully evaluate Se requirements when using a Se-deficient basal diet and supplementing with organic Se. Early studies in chicks reported that selenite was more effective than SeMet in preventing exudative diathesis [43] and raising GPX activity [44], but that SeMet supplementation resulted in higher Se concentration in breast and pancreas [43]. Recent studies in chicks report that organic Se provided as SeMet, as selenized yeast, or as the hydroxy analog of SeMet (2-hydroxy-4-methylselenobutanoic acid, HO-SeMet) are all significantly more available than inorganic Se for raising muscle Se levels. Supplementation of chicks with 0.15 μg Se/g as selenite or SeMet in a basal diet containing 0.04 μg Se/g had no significant effect on GPX activity but increased tissue Se 10–15% in liver, kidney or pancreas, and increased breast Se 30% [45]. In a study comparing selenite, selenized yeast and HO-SeMet in chicks using a basal diet containing 0.05 μg Se/g, Zhao et al. [35] found that selenized yeast and HO-SeMet supplemented at 0.2 μg Se/g increased liver GPX activity 12–26% above levels with selenite supplementation, but that there was no difference in plasma GPX activity. In these chicks, there was no effect of form of Se supplementation on Se concentration in plasma and liver, but muscle Se was increased 130% in breast with selenized yeast and 163% with HO-SeMet as compared to selenite [35]. In addition, these various forms of supplemental Se elicited significant differences in transcript expression for a few selenoproteins [35]. These reports indicate that additional studies are needed to carefully assess Se requirements in turkeys fed organic Se. Because of increased deposition of Se when supplementing livestock diets with organic Se, the European Food Safety Authority currently limits addition of SeMet or HO-SeMet to 0.2 μg Se/g diet [46,47].

This report and the previous publication collectively report the effects of dietary Se on 53 biomarkers of Se status and requirements. Use of a true Se-deficient diet showed that 0.05 μg Se/g was required to support adequate growth rate, and that there was no detrimental effect of as high as 1.0 μg Se/g on growth. New analysis of muscle, heart and kidney now shows that the minimum dietary Se requirement is >0.3 μg Se/g, and indicates that the NRC dietary Se requirement should be raised to 0.4 μg Se/g, at least for poults, to meet the nutritional need of the young turkey. The plateauing of the Se response curves from 0.4 to 1.0 μg Se/g further shows that with inorganic Se supplementation, there is but modest change of tissue Se stores with no impact on growth, indicating that this increase is safe for the young turkey. These results further indicate that the FDA limit for selenite supplementation in turkey feed can be safely raised to 0.5 μg Se/g diet. With multiple graded levels of Se supplementation from 0 to 1.0 μg Se/g, a small subset of selenoprotein transcripts were significantly decreased by Se deficiency, but no biomarkers for supernutritional or toxic Se were identified. Further studies using multiple graded levels of supplementation with organic vs. inorganic Se are needed, both in young poults as well in finishing-age turkeys, to better characterize the performance of commercial Se supplements in the turkey.

## Supporting information

S1 Table Effect of dietary Se on body weight. (DOCX)
