# Supplementary material for: Selenium requirements based on muscle and kidney selenoprotein enzyme activity and transcript expression in the turkey poult (Meleagris gallopavo)
Source: PLoS One. 2017 Nov 30;12(11):e0189001. doi: 10.1371/journal.pone.0189001 (PMC5708738; doi:10.1371/journal.pone.0189001)
Supplement: S1 Table — (DOCX) [file pone.0189001.s001.docx]

**S1 Table Effect of dietary Se on body weight**

| **Dietary Se^1^** | **Day 0** | **Day 7** | **Day 14** | **Day 21** | **Day 28** |
| --- | --- | --- | --- | --- | --- |
| (µg Se/g diet) | Body Weight (g)**^2^** | | | | |
| **0** | 59.3±1.0 | 126±1.7 | 270±15 | 413±41 | 533±60 |
| **0.025** | 63.2±1.2 | 133±1.7 | 264±20 | 408±43 | 577±57 |
| **0.05** | 65.6±1.5 | 133±9.1 | 310±20 | 494±53 | 718±95 |
| **0.1** | 63.1±2.4 | 135±6.7 | 302±11 | 467±37 | 686±68 |
| **0.2** | 62.3±2.5 | 139±7.1 | 301±20 | 519±27 | 721±37 |
| **0.3** | 60.0±3.7 | 136±6.9 | 296±24 | 460±56 | 627±107 |
| **0.4** | 64.1±1.6 | 141±2.2 | 295±12 | 527±38 | 769±90 |
| **0.5** | 60.4±2.6 | 133±7.0 | 279±25 | 451±42 | 619±64 |
| **0.75** | 66.6±1.2 | 150±5.8 | 305±21 | 506±52 | 717±76 |
| **1.0** | 64.9±1.3 | 133±2.5 | 289±9 | 473±54 | 679±127 |
| ***P-value*^3^** | *0.316* | *0.806* | *0.722* | *0.542* | *0.611* |

**^1^**Dietary Se added as sodium selenite

**^2^**Average body weights of poults supplemented with graded levels of dietary Se for 28 d (n=5/trt) and weighed weekly. Values are means ± SEM.

**^3^**P-values, as determined by one-way ANOVA, are given for each week of treatment.
